# Traditional Foods From Maize (*Zea mays* L.) in Europe

**DOI:** 10.3389/fnut.2021.683399

**Published:** 2022-01-07

**Authors:** Pedro Revilla, Mara Lisa Alves, Violeta Andelković, Carlotta Balconi, Isabel Dinis, Pedro Mendes-Moreira, Rita Redaelli, Jose Ignacio Ruiz de Galarreta, Maria Carlota Vaz Patto, Sladana Žilić, Rosa Ana Malvar

**Affiliations:** ^1^Department of Plant Production, Misión Biológica de Galicia (CSIC), Pontevedra, Spain; ^2^Instituto de Tecnologia Química e Biológica António Xavier, Universidade Nova de Lisboa, Oeiras, Portugal; ^3^Department of Genebank, Maize Research Institute Zemun Polje, Belgrade, Serbia; ^4^Council for Agricultural Research and Economics, Research Centre for Cereal and Industrial Crops, Bergamo, Italy; ^5^Instituto Politécnico de Coimbra, Escola Superior Agrária, Coimbra, Portugal; ^6^Department of Plant Production, NEIKER-Basque Institute for Agricultural Research and Development, Basque Research and Technology Alliance (BRTA), Vitoria, Spain; ^7^Department Food Technology and Biochemistry, Maize Research Institute Zemun Polje, Belgrade, Serbia

**Keywords:** *Zea mays L*., European food, flour, food quality, nutritive value

## Abstract

Maize (*Zea mays* L.) is one of the major crops of the world for feed, food, and industrial uses. It was originated in Central America and introduced into Europe and other continents after Columbus trips at the end of the 15^th^ century. Due to the large adaptability of maize, farmers have originated a wide variability of genetic resources with wide diversity of adaptation, characteristics, and uses. Nowadays, in Europe, maize is mainly used for feed, but several food specialties were originated during these five centuries of maize history and became traditional food specialties. This review summarizes the state of the art of traditional foodstuffs made with maize in Southern, South-Western and South-Eastern Europe, from an historic evolution to the last research activities that focus on improving sustainability, quality and safety of food production.

## History

Since Columbus met maize (*Zea mays* L.) in Central America in 1,492, this remarkable cereal became one of the main providers of carbohydrates in the humid Spain, Northern Portugal and many other regions throughout Europe. Nowadays, maize continues to be an essential component of the diet, particularly in America and Africa.

When the Spanish conquerors arrived at the Caribbean Islands, maize quickly became of increasing importance as the conquerors approached the mainland (1). Columbus expedition made his first bread out of cassava (*Manihot esculenta* L.), but maize was more convenient for increasing bread shelf-life. The first mention of maize bread was made by Gonzalo Fernández de Oviedo, who published the History of Indies in 1,526, including a chapter on maize (2). This new crop was called “mahiz” by Oviedo and he frequently referred to it as “*pan*,” which means bread in Spanish.

The first maize varieties used for bread in Europe came from Central America, where maize had been selected by the natives for human consumption (3, 4). Around 1,505 there is a reference to maize bread in the Mediterranean area, where the crop is compared to the already established crops in the Balearic Islands; therefore, according to Sauer (1), maize was cultivated for bread in the Mediterranean area at the beginning of the 16^th^ century. However, maize was not immediately adopted by most Europeans as food because they had wheat (*Triticum aestivum* L.) and other cereals established since ancient times and maize bread was too different from wheat bread to be easily accepted as a substitute. Therefore, maize was only used for making bread in European areas where wheat was not easily cultivated and bread makers followed the traditional European method for making wheat bread (1).

Pérez García (5, 6) finds some relationship among agricultural crisis and the establishment of maize, as farmers accept a new crop when they need to overcome a deficit that they cannot solve with the traditional crops. In Northwestern Spain, there were several recurrent crises during the 17^th^ century, due to diseases and rapid demographic growth that required new solutions for feeding people. In the coast of Northwestern Spain, maize became a popular crop after 1,630, replacing millet (*Panicum miliaceum* L.), wheat and rye (*Secale cereale* L.). In the inner areas, maize was not present at all before 1,630, and it was introduced more slowly than in the coast, following the courses of rivers. The introduction of maize in the 17^th^ century in Northwestern Spain implied some decisive changes in the agricultural and livestock system from 1,630 (7). Maize replaced millet and other cereals at different rates, depending on the location, affecting the technology, the rural economy, and several societal aspects. Maize transformed agricultural landscape and the diet wherever it became a common crop. One of the main changes was the new type of bread that was always in the family table. Actually, maize, like the replaced crops, was mainly used for bread in the coast.

In Portugal, maize was also introduced shortly after Columbus's pioneering voyage in 1,492. Ferrão (8) mentioned that it was first cultivated in Europe on the fields of Seville and was then introduced in the Portuguese Region of Coimbra. The crop fast expanded in the Center and North coast regions of the country and completely transformed rural livelihoods (9, 10), having a strong impact at technological (irrigation, fertilizing, livestock production), landscape (terraces, water mills) and social (traditions, religion, language) levels. The impact of maize expansion throughout the country resulted in a strong genetic diversification led by the crop adaptation to diversified microclimates produced by the sequences of mountains and valleys typical of the Center and Northern regions. Traditionally, the Portuguese maize bread, called “*broa*,” was made at home by women, generally once a week. Selection was made in each village for the specific preferences of the local people, which generated variability for composition, shape, size and flavor of local maize bread.

Maize was used to feed the animals and to produce maize bread that, at that time, almost completely replaced the traditional wheat or rye bread. Maize bread has become a fundamental element of the food system and regional culture. Furthermore, together with wine, maize became the currency in which rents were paid to landlords and in which farmers' wealth was measured (11). The North and Center coast regions remain nowadays the most important Portuguese areas of maize farming and traditional maize bread production, following a recipe of millet bread (*borona*) referred in Portuguese documents dating from the 12th century (12).

Maize introduction into Italy was almost simultaneous to introduction into Spain and Portugal; furthermore, since the first entrance of maize into Europe, subsequent introductions happened from the successive American territories into diverse areas of Europe, particularly in Northern and Eastern Europe. Maize cultivation in South-Eastern Europe, such as Serbia, has a long tradition, more than five centuries, starting in 1,576 (13). According to Edwards and Leng (14) maize landraces in Southeastern Europe are originated from North America, contrary to those from Spanish maize collections, which are derived primarily from Central America. The assumption was made after a natural classification of first collected maize samples in the former Yugoslavia territory. In the second half of 20^th^ century, after the introduction of hybrids, maize collecting missions were organized to collect European landraces. Collection of maize samples was followed by establishment of national genebanks in Spain, Italy, Portugal, former Yugoslavia, Romania and France (15).

## Maize Foods in Europe

### Maize Bread

Bread is a basic food of the Mediterranean diet, wheat being the main cereal used for bakery. However, in South Western Europe, whole meal maize flour is frequently used for ethnic maize leavened bread (named *broa* in Portugal) production for which the regional maize landraces (normally open pollinated varieties, OPV) are usually preferred (16–18).

The traditional “*broa*” bread making process consists of mixing more than 50% sieved whole meal maize flour, with either wheat and/or rye flour, because these cereals have gluten and, therefore, allow making bread with rheological properties similar to those of traditional wheat or rye bread. The recipe for maize bread was similar to that of wheat bread, *i.e.*, included hot water, yeast, salt, and leavened dough from a previous bread (as sourdough). The dough was then baked in a wood-fired oven. Maize bread is an ethnic product highly appreciated for its unique flavor and texture (19). Different types of maize bread can be produced by using different maize varieties and flour blendings (17, 18).

Several food technological developments have been applied to the traditional maize bread resulting in a diversification of innovative maize breads. Gluten-free maize bread, obtained by removing completely the rye and /or wheat flour in the traditional flour blending, is demanded by the gluten-intolerant people market and has been accepted in some rural communities even though its sensory quality is peculiar and has special requirements for bread making technology (20). Legume fortified maize bread formulations [by replacement of part of maize flour by legume flour, such as pea (*Pisum sativum* L.), chickpea (*Cicer arietinum* L.), fava bean (*Vicia fava* L.), or lentil (*Lens culinaris* L.)] were also well accepted by consumers (21). The optimized replacement percentages were those that allowed attaining high protein content (average increase of 21%, reaching 29% for fava bean, in relation to traditional maize bread) to label these maize breads as nutritiously enriched (21). The influence of different maize varieties (traditional-white, traditional-yellow, hybrid-white, and hybrid-yellow) and legumes on consumer liking and sensory profiling of the fortified maize breads was studied for process optimization. Chickpea flour incorporation yielded the most closely resembling sensory profile to control. These results open new opportunities for the bakery industry to develop new products to fit the growing consumer demand for healthier and high-protein food. In addition to bakery uses, maize flour has been used in different traditional recipes of maize porridge flour (with a different degree of milling) or in pastry or sweets (22, 23).

Maize became the main “bread grain” in South-Eastern Europe at the beginning of the 19^th^ century and had that primacy until the middle of the 20^th^ century in the former Yugoslavia when, with the agrarian reform of 1,957, high-yielding wheat varieties were introduced into agricultural production. This begun the period of replacing maize, as the main “bread grain”, by wheat. In just two decades, maize completely lost its role in the Serbian diet. The first written scientific study on maize as a food was published in 1,904, in Serbia; it was a report on the chemical composition of maize bread prepared in the traditional way: protein (5.7%), oil (2.2%), starch (46.2%), cellulose (1.9%), and ash (1.0%) (24). According to the rules for the quality of grains, milling and bakery products and pasta of the Republic of Serbia Regulations (Official Gazette RS, 56/2018), bread containing at least 60% of maize flour can be declared as maize bread, while mixed maize bread must contain at least 20% of maize flour. With the appearance of extended fresh bakery products in Serbia at the beginning of the 21^st^ century, the use of heat-treated maize products such as extruded maize and micronized flakes as a supplement has become popular.

The addition of heat-treated maize flour that has a pronounced ability to bind and retain water increases dough and bread yield, slows down aging and prolongs the use value of bakery products (25, 26). However, from the results, thermally treated maize flour could not improve baking quality. Substitution of wheat flour with 20% of heat-moisture treated maize starch decreased the elasticity of dough and specific volume of loaf, while the crumb containing heat-moisture treated maize starch was harder than the control (27). Due to the absence of a natural network, such as wheat gliadins and glutenins, required for holding the carbon dioxide released during the fermentation process, maize bread cannot reach the spongy texture of wheat bread. According to the results of Simić et al. (28), bread samples with 30% of maize flour showed a volume reduction of 20% compared to wheat bread. In addition, cohesiveness, resilience and springiness were reduced from 30 to 42, 40 to 53 and 16 to 20%, respectively, for maize mix-bread compared to wheat bread. Although zeins, the main storage proteins of maize (29, 30), are not able to form viscoelastic fibrils at room temperature as wheat gluten, maize flour is often used as a principal ingredient for different gluten-free foodstuffs, replacing wheat flour. As mentioned above, pure gluten-free maize bread cannot be found in Serbian markets, but gluten-free bread mixtures with the addition of maize flour and/or starch from domestic and foreign producers are sold. Gluten-free bread mixtures usually contain buckwheat (*Fagopyrum esculentum* L.), millet, rice (*Oryza sativa* L.), and/or maize flours, and guar or xanthan gums but also coconut (*Cocos nucifera* L.), tapioca (*Manihot esculenta* Grantz), amaranth (*Amaranthus caudatus* L.) and/or quinoa (*Chenopodium quinoa* L.) flours.

### Italian Polenta

The specific use of maize flour in Italy is for making the traditional dish “*polenta*,” which is very popular in Northern regions. It is a porridge-like dish, generally made by mixing maize flour with salted water through constantly stirring while heating (31, 32). The name “*polenta*” derives from earlier forms of grain mush made out of barley (*Hordeum vulgare* L.) known also as “*puls*” or “*pulentum*” in Latin) commonly eaten since Roman times. Polenta is traditionally cooked in a large copper pot, named “*paiolo*,” and is traditionally a slowly cooked dish, cooking taking sometimes 1 h or longer. Recently, instant or precooked polenta has become popular in Italy and in other countries. Polenta can be mixed (generally at the end of cooking or at the time of serving) with various foods such as butter, various cheese types, fish (in particular codfish), porcini mushrooms (*Boletus edulis*), sausages, or meat. Different kinds of polenta are prepared on the basis of the maize variety and of the type of milling. In fact, polenta is made with either coarsely or finely ground dried yellow, white or red cornmeal, depending both on the area and the desired texture (33).

### Western European Specialties: Talo and Gofio

Besides bread, maize is used in diverse recipes in western regions, “*talo”* and “*gofio”* being some of the most distinct specialties developed in Spain. The maize flour cake called “*talo*” was first produced in the 17^th^ century in Guipúzcoa (Basque Country) as a result of the replacement of wheat and barley by maize, due to soil conditions and high rainfall in that geographical area. The climate in the Basque Country and the rugged terrain made it difficult to grow wheat, so millet and chestnuts (*Castanea sativa* L.) were grown. After maize introduction, its good adaptation and productivity made that millet practically disappeared, and the local name for millet, “*artoa,”* was used to name the American cereal. Previously, bread was made of millet, chestnuts or a mixture of various flours of rye, barley and spelt, but, in 1,754, Manuel Larramendi in his “Corografía” of Guipuzcoa described the maize flour cake as the main food in the diet of the Basque population.

In this way, the maize flour cake became a substitute for wheat bread for the majority of the rural society and it was made of ground toasted maize, water and salt. Traditionally it was roasted under the ashes of the fire or on a hot metal plate called “*talo-burni”* (*talo iron*). For breakfast, pieces of maize flour cake soaked in milk were eaten; for lunch, beans with slices of maize flour cake stuffed with jerky and bacon, and, for dinner, leftovers with more maize flour cake or sweet or salty maize-based porridge. They also used oak (*Quercus sp*.) fruits which were ground and mixed with maize flour (34).

Nowadays the maize flour cake is mainly made from local varieties such as the maize called “*txakinarto,”* grain is harvested when the ear is not yet fully ripe, dried for 24 h and crushed in stone mills. Several works on agronomic characterizations of local varieties of maize in the Basque Country have been carried out (35–37). Likewise, evaluation trials of flours have been carried out for certain varieties of the Basque Country (38); the most widely used varieties for the production of maize flour cake correspond to varieties with soft and less compact starch. The composition of the flour depends on the degree of extraction–quantity of flour obtained from 100 kg of maize kernels–so as the degree of extraction increases, the proportion of starch decreases and the content of components of the cereal shells such as minerals, vitamins and fiber increases. Also the potential of the Near Infrared Reflectance Spectroscopy (NIRS) to predict the crude protein and starch content in grain and flour were evaluated in maize landraces from the Basque Country (39).

The maize flour cake is eaten wrapping a very fine and fresh “*chorizo*” or with cheese and even with sweet ingredients such as chocolate. Contrary to the arepa of Colombia and Venezuela or to the tamales or tortillas of Mexico, the maize used in Europe is not nixtamalized (maize soaked and cooked in an alkaline solution) previously; therefore, the paste does not reach a great cohesion and its texture is brittle and sandy, besides being less nutritious. In the elaboration of the maize flour cake, no yeast is added and they are cooked on the griddle, so that flat and rounded sheets of about 20 cm in diameter are obtained.

“*Gofio”* is a typical product of the Canary Islands, where maize is mainly used for the consumption of fresh ears in certain traditional foods and as a grain for the preparation of “*gofio,”* which is made with toasted flour and salt. It is usually chosen for breakfast with coffee, milk and bread. It is a product very rich in minerals, such as iron, zinc and magnesium (40). On the other hand, it is a product rich in unsaturated lipids; in particular, it has a lot of Omega 6 and a relatively low caloric content, with about 350 kcal per 100 g of product. Its fiber content makes it a food with a satiating effect (41).

The presence of maize in the Canary Islands dates back to the end of the 16th century and it is not known whether it was introduced directly from the American continent or whether it first arrived to the Peninsula and was then brought to the Canary Islands (42). Salinas-Moreno et al. (43) describes how maize and barley displaced wheat due to their better adaptation compared to wheat. In this way, the flour of the toasted maize began to be transformed in toasted flour. To make it, the maize was toasted and then milled. It seems that this food has a Berber origin, since it is a traditional food very similar to the flours used by the people of North Africa to complete their diet. Toasted flour has been produced and used as food in the Canary Islands since pre-Hispanic times. Chapter XX of the Spanish Food Register defines “*gofio”* as the product obtained by roasting flours of wheat, maize or their slightly ground grains, and subsequent crushing (44).

“*Gofio”* can also be made of tender maize kernels; the whole ear is roasted or shelled and toasted; this particular “*Gofio”* is called “*cochafisco*.” Without toasting, its flour was used to make bread. The maize “*gofio”* was usually mixed with other cereals, mainly with barley. It was consumed in the most diverse forms and accompanied by all types of basic foods and typical Canary Island desserts such as “*frangollo*” and may include raisins (*Vitis vinifera* L.), almonds (*Prunus dulcis* Mill), lemon (*Citrus limon* L.) peel and cinnamon (*Cinnamommum verum* Schaeff) (45).

The toasted maize flour can be used in many ways in the kitchen. Since ancient times, drinks were prepared soaking the flour in hot water and shaking the blend to prepare a sort of shake (46). Today it is more common to soak the flour in milk and mix it with nuts (*Juglans regia* L.). It is also used to make different types of broth such as fish or vegetable broths more consistent. Finally, the flour can be also used for making biscuits or cakes (47).

### Eastern European Specialties

Since maize was introduced as a crop in Eastern Europe, various parts of the plant have been used for human and animal consumption (24), medication (48), heating, production of bioethanol and other industrial products (49, 50). Traditional Serbian food products made from maize flour, some of which are still prepared today, include maize bread, *proja, cicvara, kačamak, komlov*, as well as drinks such as brandy *komadača* and *boza*. *Proja* is a traditional unfermented bread made from pure maize flour. Maize flour was added to a bowl of warm water until the mass is thick. A little salt and a little fat was added to the mass also. The mass was shaped and baked in brick oven. Today, unfermented bread is made with the addition of wheat flour, yogurt, sour water, baking powder, various cheeses, cracklings, herbs, different seeds, etc. The recipes of these traditional maize foods have undergone significant changes over time. Today, it is prepared as a specialty in ethnic restaurants throughout Serbia. *Kačamak* represents densely cooked maize flour in water. It is prepared in different ways depending on the area of Serbia. It can be topped with hot fat, milk, yogurt, and cheese or cream can be added to it. In addition to *Kačamak*, there are different names for cooked maize such as *palenta, puta, maljuga, mamaljiga*. *Proja* and *kačamak* are mainly made from yellow maize flour rich in carotenoids, primarily lutein and zeaxanthin. *Cicvara* is mainly prepared from white maize flour. In the boiling mixture of water, milk, cream and strong cheese, maize flour is added. The mass is continuously kneaded for a minimum of 30 min.

## Economic and Social Relevance

Economic and social relevance of maize for food has been studied in Portugal, the European country where maize for food is more relevant. In the last decades, the abandonment of agriculture in general and maize for food, in particular, was noticeable. In fact, between 1998 and 2018, the area and the production of maize in Portugal decreased 27 and 54%, respectively (51). Nevertheless, maize remains an important crop, currently representing about 2% of the National Agricultural Output; however, in 1999 that percentage was the double (52). In 2018, 714,000 tons were produced, representing 25% of total domestic use. Although the human consumption of maize has been slightly increasing in the last years, its production stays around 130,000 tons, representing no more than 5% of maize total domestic use. The main destination for domestically produced as well as imported maize is the animal feed industry (53).

The same tendency is observed in the two traditional maize bread production regions. In the Northern (North coast) region, between 1998 and 2018, the area and the production of maize were reduced 56 and 52%, respectively (51). In the 1980s and 1990s farmers largely replaced maize for human consumption by maize for animal feed. However, more recently, small farmers have also abandoned livestock farming and the small (<0.5 ha) and unprofitable plots have been left unproductive. In the Center coast region, this trend, although visible, is less pronounced. The area and production of maize diminished 49 and 10%, respectively, in the same period. A progressive decrease in the production and use of traditional varieties also occurred and, at the same time, the traditional knowledge related to the selection of varieties and the production of maize bread was neglected (54). Nowadays some traces of the traditional practices remain but only in household production, for family consumption and direct selling.

Against this scenario, and having in mind that “*broa”* is a public good, a cultural heritage resulting from empirical evolution over many generations, some collective actions have been developed at local level aiming at the definition of marketing plans able to increase maize bread visibility and use it as a lever for sustainable rural development. Maize for food is a crop that most farmers are familiar with and could be valued when used in regional traditional maize bread production. The traditional varieties have excellent characteristics to be used in bakery, supporting the development of the maize bread value chain.

However, traditional varieties are less productive and more susceptible to climatic stress and diseases and therefore farmers have replaced them by more productive varieties, unless their families bake maize bread for their own consumption. In a case study developed in the Northern region (54) this problem is identified by local actors as one of the most significant threats to the success of an initiative aiming at the development of sustainable maize for food supply chain. It also shows that the main incentive to encourage farmers to produce maize varieties suitable to bake traditional maize bread is a higher price for maize traditional varieties. The case study also indicates that final consumers as well as restaurants seem willing to pay a price premium to reward farmers for the lower yield associated with traditional varieties. At the processing level, local actors raised the question of legislation as one of the major threats for maize bread, arguing that, in Portugal, European rules on food safety are not realistically applied to endogenous food products. A label for traditional maize bread could support local development, similarly to what currently happens with wines and other certified products, not only because it may enhance the maize value chain, but also because it may produce positive externalities in other sectors, namely gastronomy and tourism. The growing interest of some new farmers in endogenous products, as well as the increase in demand, are considered by local actors as opportunities for initiatives aiming at the establishment of a label for local traditional maize bread.

## Maize Food Quality and Genetic Diversity

The first maize varieties introduced from America were mainly flint, and they were the base for the subsequent adaptation and selection for diverse uses; actually, local consumers still prefer flint grains of several colors (white, yellow or black), though white grains are normally used for bread, while yellow is used for feed. Traditionally, most people prefer white flint maize for food and particularly for bread because it produces breads more similar to wheat bread. Nevertheless, colored maize contains antioxidants that have interest as functional food (55, 56).

Based on our own experience, autochthonous maize populations are growing in importance. Increased interest in populations is derived from the need of reverting the negative consequences of modern breeding for increasing productivity under high inputs, which has reduced the genetic basis and narrowed diversity and, consequently, could favor the loss of beneficial genes for grain quality and stress responses. Traditional maize populations and varieties are a potential source of traits for increasing the nutritional and functional value of grains, since the content of carotenoids, phenolic compounds and antioxidant capacity in maize grain is higher in relation to wheat, oats (*Avena sativa* L.) or rice. Colored grains, *e.g.*, yellow, orange, purple, blue, or black, are rich in bioactive phytochemicals, i.e., numerous secondary metabolites, and receive more and more interest nowadays.

Maize germplasm in South Western Europe shares origin and history, as well as traditional uses for bread. Revilla et al. (57) assessed agronomic performance and baking quality among autochthonous maize varieties and identified those with the best performance under organic conditions and quality for making bread and traditional maize foods. The selected varieties were *Tuy* (yellow kernel and medium growing cycle), *Sarreaus* (yellow kernel and early cycle), *Meiro* (black kernel and late cycle), and *Rebordanes* (white kernel and medium-early cycle). Garzon et al. (58) assessed significant diversity among maize populations from Spain and the United States for dough rheology and gluten-free breadmaking performance, and reported that endosperm type affected bread crumb color, dent maize having higher color parameters and the rheological parameter called “instant recovery speed.” These authors also reported that population origin affected the quality parameters flotation index, onset pasting temperature, bread crumb color, hardness and instant recovery speed. Furthermore, growth cycle affected flotation index, crumb color and cohesiveness. Water-binding capacity, crumb color and hardness were the most discriminative parameters for maize. Though all maize accessions produced bread well differentiated from wheat bread, Garzon et al. (58) concluded that the maize population *Andaluz/Daxa* was the less distant from wheat for many parameters, and the population *Tremesino* was the most distant. Moreira et al. (59) found no significant diversity among maize varieties of white, yellow and purple grain for water desorption isotherms; however, white maize flours showed higher average particles size than purple and yellow maize flours.

In Portugal, a unique germplasm has been developed through centuries of adaptation to local environment and food uses, in particular, for the ethnic maize leavened “*broa”* bread production (60). Because of their use for human consumption, the national maize landraces are in part maintained, and not yet totally replaced by commercial hybrids (17). In the beginning of the 21^st^ century more than 50 traditional maize varieties, still found under cultivation, were collected directly from farmers from a typically maize-based bread-producing traditional region (17). The 50 Portuguese varieties evaluated by Vaz Patto et al. (17) were characterized regarding their flour's quality, and agronomic performance, complemented more recently with a genetic diversity and genetic structure analysis (18, 61). Flour obtained from each population was used to study flour basic nutritional composition, flour's pasting behavior, and bioactive compounds concentration. Most farmers' populations analyzed showed high levels of protein and fiber, low levels of carotenoids, volatile aldehydes, α and δ-tocopherols, and low breakdown viscosity values (61). In farmers' populations, large variation was found for flour yellowness and total carotenoids, and for the two individual phenolic compounds analyzed p-coumaric acid and ferulic acid (61). This indicates that further improvement to increase the attractiveness of food formulations based on these populations, and specifically for those traits, where variation can still be found, is possible. Accordingly, the Portuguese traditional maize varieties preferred by local consumers for bread, such as *Fandango* and *Pigarro*, showed significant higher protein and lower amylose content than hybrids (20).

Historically, Italian maize polenta was obtained from landraces from the different microclimates specific of the several Italian agroecological and agroclimatic areas (62, 63). Zeppa et al. (64) reported that quantitative descriptive analysis can be used for sensory description of polenta produced with the simple recipe (maize flour and water), and that the defined lexicon (13 terms: four for odor, three for taste, four for aroma and two for texture) can be used to describe the sensory qualities of polenta produced with 12 Italian maize cultivars. In the first large screening of Italian maize germplasm, 633 landraces were characterized by NIRS for their kernel composition (content of protein, lipid and starch, floating area) and compared to 519 landraces from 20 different countries (65). The carotenoid total content and composition (lutein and zeaxanthin) of the 93 maize landraces of the European Maize Landraces Core Collection (EUMLCC) were also determined. The screening revealed the presence of a wide genetic variability for all traits. The set of Italian landraces comprised in the EUMLCC showed a higher content of total carotenoids as compared to landraces from other countries, and zeaxanthin was more abundant than lutein (on average, 10.46 vs. 5.89 mg kg^−1^ dm). As a result, Italian landraces *Nostrano dell'Isola* and *Marano*, characterized by high percentages of protein, lipid and carotenoids and a vitreous texture, were considered interesting in breeding programs and were introduced into high-yielding, modern genotypes, with the aim to be exploited by the food industry. This peculiar carotenoid composition of Italian germplasm, *i.e*. a larger content of zeaxanthin than lutein (on average, 11.77 vs. 7.13 mg kg^−1^ dm), was confirmed in another study, which reported a detailed characterization of carotenoid fraction in a set of Italian and public inbred lines (66). Cryptoxanthin, being a precursor of zeaxanthin, was also quite abundant (2.47 mg kg^−1^ dm). In general, the Italian inbreds, selected from traditional maize populations that are still appreciated for their organoleptic properties, are also suitable material for improving nutritional quality of modern maize hybrids for the market of traditional maize-based foods.

Comparison of maize hybrids and autochthonous landraces from Eastern Europe (Serbia and Bosnia and Herzegovina) maintained at the Maize Research Institute Zemun Polje gene bank, differing for grain color, physical properties, grain protein fractions, phenolic compounds, carotenoids content and antioxidant capacity resulted in higher values obtained in landraces (67). Autochthonous varieties have better technological grain quality and performed better than hybrids for dry grain milling and snack food production. Local landraces represent a pool of valuable grain quality traits, as well as a source for grain improvement in commercial hybrids. Ten landraces and inbred lines, with various grain colors were evaluated for total phenolics, flavonoids, anthocyanins, β-carotene, lutein, free, conjugated, and insoluble bound phenolic acids and antioxidant activity (68, 69). Significant variability in phytochemical contents and antioxidant capacity were detected; for example, the content of lutein and zeaxanthin in yellow maize genotypes of the Serbian collection varies from 5.9 to 13.9 μg/g (68, 70). Inbred line with orange kernel was superior in β-carotene content (2.42 mg/kg d.m.), while the inbred with light blue kernel has a higher content of total phenolics (5778.2 mg GAE/kg d.m.), flavonoids (337.5 mg CE/kg d.m.) and ferulic acid (3274.6 μg/g d.m.). Although local landraces are dynamic populations, result of natural and human selection, adapted to particular growing conditions, introduced materials may be also very valuable, containing traits not existing in locally-adapted material (71). In the Serbian collection, oil, protein and starch contents were significantly higher (0.43, 0.12, and 0.85%, respectively) in introduced populations than in local landraces. A subset of local and introduced populations from a drought core collection (25 in total), were analyzed for protein, oil, starch and tryptophan contents. Starch and oil contents were 0.84 and 0.39%, respectively, higher in introduced populations. Besides, SSR (Simple Sequence Repeat) primers for *opaque2* recessive allele (*o2*) showed that most accessions (23) had high tryptophan content, and in most of them the recessive *o2* allele was found (72). Correlations between proteins, tryptophan and oil make some accessions suitable for simultaneous multi-trait improvement through breeding. These high quality accessions with drought tolerance are good source for nutritional improvement of maize grain.

## Research and Breeding Focuses

### Nutrition and Health

Maize food, such as bread, is a healthier alternative to wheat bread due to its lower glycemic index (19). *Broa* was considered by CNN Travel (73), in October 2019, one of the 50 world's best healthy peasant breads. As a cereal, maize is not a balanced food for humans; though whole grain is a source of dietary fiber and minerals, other vegetables are richer in dietary fiber and minerals contents (74). Besides carbohydrates, maize provides minerals, vitamins and antioxidant compounds, and has high content of potassium, sodium, chlorine and sulfur. Maize protein is poor in lysine and tryptophan but rich in methionine and cysteine.

A second relevant aspect of maize for food is that maize lacks gluten and, thus, it is a convenient food for people suffering from celiac disease, which is an enteropathy caused by ingestion of wheat gliadins and prolamins from rye and barley (75). Those proteins induce an autoimmune response by the activation of IgA antibodies (76, 77). Gluten is actually a big problem for celiac patients because even traces of gluten can seriously affect them; therefore, very sensitive methods are required for detecting residues and even the most efficient methods for removing gluten cannot guarantee the complete safety of food for celiac patients if wheat or other cereals with gluten are used in the same facilities (78). Torres et al. (79) published a review of bioactivity of available gluten-free food products made with cereals [rice, maize, sorghum (*Sorghum bicolor* L.), teff (*Eragrotis curvula* Zucc.) and millets], pseudocereals [buckwheat (*Fagopyrum esculentum* Moench.), quinoa and amaranth] and other food crops. Unlike wheat gluten, according to numerous studies, maize zeins do not contain toxic and immunogenic peptides. However, hypothetically, maize zeins could be harmful for a very small group of celiac disease patients, especially those that are nonresponsive (80).

Maize can be also a functional food because of pigments and related metabolites with antioxidant activity. Indeed, carotenoid, xanthophyll and anthocyanin have been associated with a reduced risk of degenerative diseases including cancer, cardiovascular disorder and impaired vision (55, 56). Orange and yellow maize contain carotenoids, being β-carotene the carotenoid with the highest pro vitamin A activity (81). The most abundant carotenoids in maize grain are lutein and zeaxanthin, and carotenoid content is regulated mainly by additive effects, which implies that they can be improved by selection (82).

Among cereals, pigmented maize is the most important source of anthocyanins and it can be a substitute for wheat in cookies and crackers both because of the attractive color and the healthy effect. In order to preserve the stability of phenolic compounds and color formation in dark red and blue maize cookies, Žilić et al. (83) added 0.2 and 0.4% of citric acid to the mass, which contained colored maize flour, refined palm oil, sucrose powder, nonfat dry milk, sodium bicarbonate and water. Blue popping maize cookies with citric acid had pH values of 4.41 and pink color. Lightness (CIE L^*^) and redness (CIE a^*^) of blue popping maize cookies with citric acid were increased compared to colors of their corresponding doughs. The results of these authors show that citric acid had a stabilizing effect and improved accessibility of the anthocyanins. The content of anthocyanins in the blue popping maize cookies with 0.2% of citric acid increased by 1.11-fold in the flour. Reduced pH with citric acid at low temperatures restricted Maillard reaction and browning during baking of maize cookies. At pH 7.39 to 7.85 in yellow, red and blue maize cookies, Kocadagli et al. (84) found a high content of 3-deoxyglucosone, as well as higher sucrose hydrolysis. However, these authors state that colored maize flour could be the source of natural dietary antiglycation agents due to good abilities of their phenolic compounds to trap C_2_, C_3_ and C_4_ α-dicarbonyl compounds. The effect of maize flour and baking conditions on the formation of Maillard reaction products in cookies was studied also by Žilić et al. (85). Red popping flour with the lowest content of free asparagine of 189.7 mg/kg, generates the least acrylamide in the cookies in relation to white, yellow and blue maize flours. The results also show that the acrylamide content after 13-min baking was higher in cookies made of hulless oat, durum wheat and rye flours than those made of maize flours.

The development of new pigmented maize genotypes rich in bioactive (antioxidant) compounds could be a good tool to increase the functional properties and healthy power of the human diet. Italian local varieties from the CREA Genebank in Bergamo (86, 87) were crossed with germplasm from Bolivia (purple “*Morado*"-type) and from Mexico (blue “*Azul*"-type) and selected to obtain new pigmented maize genotypes, in the frame of the International Cooperation Project P.S.G.O. Km 0 Bolivia (2018–2021) (88). The materials developed in this research could be valorized also considering that, as reported by Rodríguez et al. (16), the selection for red kernel color intensity in maize, yields high levels of anthocyanins that vary from black maize kernels to white grains from the same variety and produces higher antioxidant activity in the kernels with darker color. The development and study of maize cultivars rich in anthocyanins for colored polenta as new functional food were also reported in literature (89, 90).

Pigmented maize can be a beneficial food for humans due to the presence of numerous antioxidant substances and bio-functional compounds. These compounds are present in the whole kernel in different concentrations depending on the pigmented maize genotype (91). The results reported by Lago et al. (89) showed that anthocyanins content of maize flour decrease of about 22% in the anthocyanins content of polenta probably due to the cooking process, as previously highlighted in maize by Salinas-Moreno et al. (43) and in fruits and vegetables by Jones (92). Nevertheless, based on previous reports, despite the decrease due to cooking, the colored polenta retained appreciable amounts of anthocyanins and good relative levels of antioxidant activity, and can be considered a functional food. Additionally, the taste perception by consumers revealed no differences between colored and uncolored polenta (89), suggesting that selection of new maize polenta varieties, rich in anthocyanin pigments, could be an acceptable option for consumers and a valuable tool to increase the antioxidant power in human diet.

The significant maize variability for grain composition is source of favorable alleles for improvement of grain micronutrient content by conventional breeding or by biotechnology, for better utilization in human diet. Therewith, colored grains, rich in anthocyanins, have various health benefits, preventing diseases associated with oxidative stress. In the investigation of composition and content of anthocyanins in the grain of blue popping maize, deep purple maize, purple wheat, and black soybean (*Glycine max* L.), the following results were obtained: deep purple maize had the highest content of total anthocyanins (4988.9 mg cyanidin 3-glucoside equivalent/kg d.m.), with dominance of the glucosidic forms; ten anthocyanins were identified in blue popping maize and the acylated forms of anthocyanins were dominant. The study also highlighted colored maize as one the best sources of anthocyanins (93).

Currently, given the specific requirements of today's consumers in Serbia, wholegrain flour of specific maize genotypes, such as open pollinated varieties, rich primarily in bioactive compounds, is added to the wheat flour in order to increase the functional value of bread. Replacing 30% of wheat flour with wholegrain blue and dark-red maize flour increased phenolic compounds content in bread and its color (28). The same authors reported that the content of total phenolic compounds and ferulic acid was from 2.8 to 4.2-fold and from 22 to 25-fold, respectively, higher in maize mix-breads than in wheat bread. Despite thermal degradation, the content of total anthocyanins in crumb of blue and dark-red maize mix-breads was relatively high and amounted to 142.3 mg cyanidin 3-glucoside equivalent/kg and 84.4 mg cyanidin 3-glucoside equivalent/kg, respectively, which is a considerable enrichment of wheat bread in terms of its functionality and health effects.

Nutritive value of maize bread can be improved by adding ingredients, for example, Collar et al. (94, 95) reported that blended maize matrices with added resistant starch and wheat flour modified textural profile, crumb grain features and firming kinetics, and free polyphenol pattern of breads compared to the maize bread alone. All maize breads had high-fiber, and enriched bread at large provides enough resistant starch to positively affect postprandial glucose and insulin levels and enhance health.

Comparing to other cereals, maize contains a high level of soluble phenolic compounds, in particular of hydroxycinnamic acids such as the free ferulic and p-coumaric acids (96). These phytochemicals are considered health promoting components, with anti-bacterial, anti-aging, anti-carcinogenic, neuro-protective, cardiovascular and anti-diabetic properties (96). Interestingly, the major soluble phenolic compounds present in maize flour, hydroxycinnamic acid amides, particularly dicoumaroyl spermidine, coumaroyl feruloyl putrescine and diferuloyl putrescine, resist to maize flour fermentation and baking process. Furthermore, using traditional processing conditions, the bread content in free ferulic and p-coumaric acids increased more than 3-fold compared to flour contents, meaning that the bioaccessibility of maize phenolic compounds could be improved, substantiating the potential health benefits of *broa* (97).

In order to implement the scarce references about phenolic compounds in yellow maize, a simple and repeatable method for spectrophotometric analysis of soluble phenolics content (SPC) in maize flour was used, and a first screening of 81 genotypes, comprising inbred lines, landraces and F1 seeds, was carried out (98).

Total antioxidant capacity (TAC) represents an important tool to estimate the potential pro-healthy value of a raw material. TAC can be determined by different enzymatic methods (99–101). A chemometric model for the prediction of TAC in maize flours by NIRS was assessed in a set of 391 samples, comprising Italian and public inbred lines, F1 seeds, commercial hybrids and traditional varieties, using ABTS direct assay as reference value (102). The good stability of the regression coefficients allowed developing both global and specific predictive models for this trait, demonstrating that NIR spectroscopy could be applied efficiently to the screening of the genotypes.

Maize is widely considered a “key” raw material in gluten-free (GF) formulations (103, 104), as maize starch is a “viscosity-builder” through the gelatinization and retrogradation phenomena that occur during the processing of GF foods (105). The increasing request for maize for food production stimulated the identification of Italian genotypes having specific qualitative parameters, including kernel hardness and peculiar starch properties, both strategic for food processing (106).

Besides diversity available among natural genetic resources, there are several natural mutants that can increase the genetic resources for maize breeding, for example, Revilla et al. (107) evaluated the bread potential of high amylopectin (*waxy1*) and high-quality protein (*opaque2*) maize mutants for bakery and found that while *o2* had no significant mean effects, *wx1* decreased flotation, onset pasting temperature, maximum and final viscosity, trough, breakdown, total set back, area under the apparent viscosity curve, hardness, chewiness and instant recovery speed, and increased water binding capacity, reddish crumb color, retarded recovery elasticity and cohesiveness.

### Safety

One of the major safety problems of cereals is the contamination with mycotoxins, as the European Mycotoxin Awareness Network and FAO recognize (108, 109). Mycotoxin contamination of maize grain is a global threat to safety both for human food and animal feed (110). Mycotoxins are secondary metabolites produced by toxigenic fungi, which may be toxic or have other debilitating effects on living organisms (111). The chronic exposure to mycotoxins represents a critical factor for human health (112).

Mycotoxins are fungal metabolites that, when ingested, inhaled or absorbed through the skin, cause low performance, sickness or death in man or animals. They are produced by fungi of genera such as *Fusarium, Aspergillus* or *Penicillium*. There are more than 300 mycotoxins with diverse chemical, biological and toxicological characteristics that can be produced in many crops and derived products. Mycotoxins and their derivatives have diverse toxic potential, depending on the type of mycotoxin, concentration, length of exposure and characteristic of the exposed individual, and damage can be especially important on liver, kidneys, and immune, endocrine or nervous systems (113). They can be mutagenic and carcinogenic; potential carcinogenic risk for some mycotoxins has been rated by the International Agency for Research on Cancer (IARC). Therefore, legislation to limit the amount of some mycotoxins has been implemented in many parts of the world to minimize human health risk. Accordingly, several international institutions have made recommendations and stablish concentration limits (114), which are particularly strict in the European Union (115). The incidence and severity of mycotoxin contamination in Italian maize cultivated area, mostly caused by *Fusarium verticilliodes* (fumonisins) and by *Aspergillus flavus* (aflatoxins), is highly dependent on genotype, agronomic practices, environmental conditions, biotic and abiotic stresses (116–118).

Combination of vegetal products can be useful for mitigating mycotoxin damage. Fumonisins can be controlled with isothiocyanates that are natural compounds from plants of the *Brassicaceae* family, produced by enzymatic conversion of glucosinolates and decrease *Fusarium mycotoxigenic* strains by reducing mycelium size (119). Isothiocyanates also reduce fumonisins levels naturally produced in bread by *Gibberella moniliformis* and bioaccessibility and bioavailability of fumonisin (120).

Fermentation and baking of dough also cause important mycotoxin losses (121, 122); therefore, bakery improves the safety of maize foods. However, this process is the objective of several researches. Krska et al. (123) carried out the project MyToolBox for developing novel interventions aimed at achieving a significant reduction in crop losses due to mycotoxin contamination. Among the diverse focus of that project, they also studied the effects of baking on mycotoxins at an industrial scale. Lino et al. (124) analyzed breads from Northern Portugal and 73 % of samples had fumonisin levels above the limit established by the European Union, though they were not considered a health hazard because of the low proportion of bread in the diet. Similarly, breads from different regions of Portugal have diverse concentrations of ochratoxin A depending on the characteristics of the bread and the regions (125, 126). Ochratoxin A (OTA) is a secondary fungal metabolite produced by *Aspergillus ochraceus, A. carbonarius, A. niger* and by *Penicillium verrucosum* that can be present in most bread samples of the Atlantic coast (127).

Maize kernels can be contaminated with mycotoxins mainly at the pre-harvest stage (128), and infestation is promoted by high temperature and insect damage (129). Cao et al. (130) investigated environmental factors related to fungal infection and fumonisin accumulation during the development and drying of maize and reported that *Fusarium verticillioides* was the most prevalent species. They also found that temperature and moisture conditions during the first 80 days after silking favored natural kernel infection by *F. verticillioides*. Fumonisin was found in kernels 20 days after silking, though fumonisin accumulation was not above levels acceptable in the EU until physiological maturity is achieved.

One of the factors involved in increasing fumonisin was insect damage, particularly *Sitotroga cerealella*. The main insect pests of maize in Southern Europe are the corn borers *Sesamia nonagrioides* and *Ostrinia nubilalis* (131). Butrón et al. (131) evaluated corn borer damage in northwestern Spain and found that the Italian late white variety *Bianco Perla* was the most appropriate to obtain high yield along with reduced stem damage by *S. nonagrioides* and reduced risk of fumonisin contamination, along with the early Spanish varieties *EPS12(T)C3* and *Rebordanes*. Other mycotoxins are not detected in maize bread; for example, Herrera et al. (132) developed a straightforward analytical method to determine the mycotoxin moniliformin in cereal-based foods and reported that moniliformin was not detected in bread samples (133).

The Italian maize germplasm maintained at the CREA Genebank in Bergamo contains nearly 500 inbred lines and 600 local varieties (landraces) gathered in different regions in the 50's, when the cultivation of hybrids begun to was replace landraces; so, it represents an interesting starting point for searching genotypes resistant to fungal pathogens useful to reduce mycotoxin contamination (87, 118). The screening through field artificial inoculation of inbred lines (118) and traditional varieties (87) highlighted that there are some interesting maize Italian germplasm sources of resistance to mycotoxin accumulation useful to support grain quality safety. On the other hand, 240 inbreds from the Spanish collection, of different origins, were evaluated in the field for 2 years for grain resistance to *Fusarium verticilloides* and fumonisin with artificial inoculation. Sixty-one inbreds were found to have the highest levels of resistance to both across years (134). Inbreds differing in kernel color, use, kernel type and heterotic group were all represented in this group of the best inbreds. Many of these inbreds are being used in breeding programs to improve *F. verticilloides* and fumonisin resistance (134).

Research reported in literature indicated that pigmented maize kernels could express possible plant defense functions against biotic (fungal pathogens and pests) and abiotic (drought) stresses because of the high antioxidant power of components determining kernel pigmentation (135–138). A recent study highlighted the role of phlobaphenes (insoluble phenolic compounds conferring a red-brown pigmentation to kernel pericarp) in modifying pericarp thickness and reducing fumonisin mycotoxins accumulation (139). The traditional Italian cultivar *Nero Spinoso* from the Camonica Valley had a very high level of phlobaphenes and a pericarp layer thickness significantly higher than the colorless control (140). In this perspective, maize pigmented genotypes developed for their content in specific bioactive phytochemical compounds, could be also suitable to be introduced into advanced breeding programs aimed to enhance and valorize biodiversity potential on resistance to mycotoxigenic pathogens and on grain quality and safety improvement. In the evaluation of the inbred collection from the MBG germplasm bank, white corn inbreds had higher levels of fumonisin than yellow ones, but it was still possible to find white inbreds with comparable resistance to fumonisin accumulation to that of the most resistant yellow inbreds (134).

Other health hazards include metals, such as Aluminum, whose toxicity restricts cultivation of crop plants and causes substantial losses of production in areas with acidic soils. Garcia-Oliveira et al. (141) stated that maize is relatively less sensitive to Aluminum contamination. Other metals are not a major problem in the areas where maize is used for bread in Europe.

### Quality

The first Europeans accepted maize as food when they were hungry, because, in the 17th century, maize was considered nurture for vulgar people (142). Quality parameters have not been specifically defined for maize bread; though, FAO (143) classified five quality grades, based on grain density, proportion of whole grains, damaged grains, and grain color. High quality implies high test weight, low proportion of broken grains and foreign material and color uniformity. Moreira et al. (59) developed a model to predict flour color involving color parameters of the different particle size fractions. Flint maize is hard and produces large particle size that made flour rougher and reduce reflectance (144). Grain density is important for dry milling but reduces yield (145).

In what concerns the technological ability of Portuguese maize for maize bread production (61) (*breadability*), several parameters related to kernel composition, flour pasting behavior and flour particle size have been previously identified as crucial (20, 146). Brites et al. (20), through a sensory analysis on maize bread carried out by a trained panel with different maize varieties, identified a preference for maize bread produced using open-pollinated populations, due to texture, taste, and aroma, as opposed to maize bread produced using commercial hybrid maize varieties. Furthermore, the flour from open-pollinated populations, besides having better quality maize bread, had higher values of protein, lower values of amylose, and lower viscosities (maximum, minimum, final, and breakdown viscosities) (20). More recently, technological processing quality differences between the higher-yielding dent hybrids and the hard endosperm Portuguese open-pollinated populations have shown that the maize bread produced with the dent hybrid varieties had higher specific volume; however, a sensory analysis showed a preference for the maize bread made using Portuguese open-pollinated populations due to better mouthfeel flavor and texture (146). The higher protein contents detected on the varieties with higher *breadability*, can probably induce increased amounts of water absorption ratio by the flour enlarging bread moisture. In fact, the crumb moisture was identified (146) as a relevant attribute for consumer acceptability of maize bread. As maize lacks gluten, obtaining the spongy texture in bread made with maize is a technological challenge because maize lacks the natural network required for holding the carbon dioxide released during fermentation (147). Brites et al. (20) also evaluated different milling processes (attending to formulation and processing variables) on quality attributes of maize bread and concluded that grinding in a water mill takes more time, had lower ash content and higher maximum, minimum and final viscosities than in electrical mill. Traditional processing allows damage by insects or fungi that are dangerous for human health (148). Though the effect of most environmental factors, such as plant biodiversity or pollination quality, is poorly known, bread quality depends on environmental and genotypic factors such as grain weight, moisture, uniformity, density, and lack of physical, disease or pest damages (149).

As fermented food, sugars are converted into organic acids in maize bread, affecting organoleptic, rheological properties and self-stability (150, 151). Other effects of fermentation are increase of viscosity and crumb firmness (152) as well as other physical and chemical modifications. Pico et al. (153) found that fermentation maintained stable levels of hexanal, hexanoic acid, benzaldehyde, benzyl alcohol, furfural and furfuryl alcohol but increased other volatile compounds. Conversely, baking evaporated 2,3-butanedione, 1-propanol, 2-methyl-l-propanol, 3/2-methyl-1-butanol and ethyl octanoate and increased other volatile compounds. The main volatile compounds in dough were the alcohols from fermentation, 2,3-butanedione, acetoin, acetic acid, isobutyric acid and ethyl octanoate, and, in crumb, also hexanal, 1-octen-3-ol and nonanal were important contributors.

Conservation of quality is another important factor for perishable foodstuff. Revilla et al. (154) assessed the influence of growing and storage conditions on bakery quality of maize for bread under organic agriculture. They studied the genotypic and environmental effects on grain quality for human consumption using traditional grain processing methods. Genotypic effects were more important for grain quality; meanwhile environment was more important for grain yield and pericarp damage. Warm-air drying increased grain quality and medium-term storage increased grain quality but reduced germination percentage. The main factors for optimizing quality were appropriate growth cycle and proper harvest time. Furthermore, quality and agronomic performance can be managed independently of each other except for the negative correlation between moisture and milling test.

Bread attributes depend also on physical characteristics, such as flour particle size (155). De la Hera et al. (155) found that finer-particle-size flours lowered dough development during fermentation in all cases, flours with compact particles had higher specific bread volume and greater particle size, dough with more water gave breads with higher specific volume, especially in compact flours. They concluded that flours with coarser particle size are the most suitable for gluten-free maize bread. Other methods of improving quality include mixing flours from different cereals; actually maize and rice can be used for gluten-free food because both cereals lack gluten. Mancebo et al. (156) studied quality of mixtures of gluten-free flours and found that incorporating starch increased bread specific volume and cell density, but not color, and quality was worse compared to wheat bread.

### Niacin Availability

Maize content in niacin is not naturally available for humans without adequate processing, and lack of niacin causes pellagra. The introduction of maize into European diet was associated with specific nutrient deficiencies such as pellagra, a disease that was unknown in Europe due to the nutritional value of the European cereals (157). Pellagra is the result of a diet that does not contain niacin (vitamin B3) as well as tryptophan, its precursor. Pellagra was common in some regions of Europe, and still is today in sub-Saharan Africa, in people who mostly subsist on maize. Symptoms of pellagra include inflamed skin, diarrhea, dementia, and sores in the mouth. Anorexia and malabsorptive diarrhea lead to a state of malnutrition and cachexia and, without treatment, may result in death (158). In America, this deficiency was solved with a process called nixtamalization, which is an alkaline solution treatment which corrects the niacin deficiency, and was a common practice in Native American cultures. Žilić et al. (70) indicated that 8–65% of total niacin was liberated from a chemical linkage by alkaline treatment. Tryptophan-rich maize had a lower content of total niacin (99.44 mg/kg) and higher content of available niacin (42.61 mg/kg) than regular maize. The earliest description of pellagra was made by the Spanish physician Don Gaspar Casal in 1763 (159). Pellagra spread through Europe from Spain to Italy, France, central Europe, Romania, Turkey, Greece and parts of southern Russia (159). For over 200 years, between the 18th and the 20th centuries, pellagra was endemic in those parts of Italy that depended on maize as a staple crop (157). Pellagra affected over 100,000 people in Italy by the 1880s, when it was attributed to the extensive consumption of maize as “polenta” (160). During the late 19th century, hypotheses emerged that maize either carried a toxic substance or was a carrier of disease since pellagra outbreaks occurred in regions where maize was a dominant food crop. Later, researchers found that pellagra could be prevented by modifying processing techniques or by including animal and leguminous proteins or a small amount of brewer's yeast.

### Breeding Programs

The market sector of maize for bread is limited compared to markets for other major uses of maize. Consequently, few specific breeding programs to improve maize varieties for bread have been developed in Europe. The most outstanding project is the VASO project in Portugal that started in 1984 by Silas Pêgo and is an on-farm participatory maize breeding (PPB) project at the Portuguese Sousa Valley region (9, 10, 18). The breeding program focuses on yield, bread making quality and the ability for polycropping systems. This breeding program was applied to local varieties as *Amiúdo* or *Pigarro* and to a synthetic variety named *Fandango* that represents a transversal project between on-station and on-farm programs. The project carried out with *Pigarro* (161) includes two selection programs i) phenotypic recurrent selection and S_2_-lines recurrent selection (named breeders selection) and ii) stratified mass selection with two-parental control in three sequential steps (two in the field and a third one at the storage facilities) (named farmers selection) (162). A joint molecular and agronomic comparative study that took place under this PPB program using *Pigarro* (162), indicate that for both farmers and breeders' selection methods, genetic diversity was maintained, even with the more intensive breeder's selection, suggesting that further response to selection can be expected. However, yield increase was only detected during farmer selection. Indeed, farmers' selection was more effective in increasing fasciation-related traits and cob weight, what might have contributed for the yield increase. Ear fasciation is particularly important for farmers, to maintain a certain level of diversity, toward a long-term gain in ear diameters, kernel-row numbers, medulla and rachis dimensions (17). The breeders' selection on the other hand, was more effective in achieving crop uniformity, plant and ear height reduction and greater resistance to stalk lodging (162). So, although both selection approaches achieved phenotypic modifications though preserving genetic diversity, only the farmers' selection resulted in a yield increase and, as it is a cheaper methodology, technically more accessible to farmers, this would be the choice for future PPB.

Besides comparing the two selection approaches applied in the Portuguese maize PPB program, the impact of the breeding activities on the maize populations' agronomic performance improvement has been also measured in four out of the several maize populations in the program (161, 163, 164). Furthermore, the temporal changes in genetic diversity were evaluated for three of those populations (163, 165). Overall, participatory breeding methodologies were partially effective in improving the agronomic performance of the maize populations, while maintaining high molecular diversity in these populations. The quality traits evolved randomly across the breeding program (163). This observation warns for the need to develop selection tools for characteristics that cannot be visually selected by farmers. Indeed, a more detailed evaluation of the effect of breeding programs on quality aspects is needed since the quality of these genetic resources for maize bread production is a decisive aspect for the on-farm maintenance of these historical populations and for their present market added-value recognition (20, 60). VASO project updates have been able to include transdisciplinary and multifactor approach that includes farmers and rural development associations, millers, bakers, academia, municipalities and consumers defining targets in the network filling the gaps from the seed to fork; searching economic models that have to be created and adapted. VASO project seeks to improve germplasm and its use so that varieties are attractive to consumers, the processing industry and farmers, responding to public concerns related to health and the environment, increasing the sustainability of agricultural systems and contributing to the short chain and well-being of farmers, for which a greater involvement of national and local actors (GO) are needed (54). It is also important to stress that VASO paved the way to promote use and valorization competitions such as the “Best ear of Sousa Valley” in which development of genetic resources and pedagogical tools based on statistical tools were elaborated and made available to farmers (166). Another breeding program was carried out in the Basque Country, Spain, by Ruiz de Galarreta from the “Instituto Vasco de Investigación y Desarrollo Agrario” NEIKER in collaboration with the Federation for Organic Agriculture of the Basque Country (North of Spain) for improving the *talo* (a special type of maize bread) quality of the variety Donostia (38).

Breeding maize for bread can also focus in a specific target, such as improving the value as functional food by increasing the antioxidant activity; for example, Rodríguez et al. (16) evaluated a selection program for color intensity on antioxidant capacity in maize. They reported that pigment content was directly related to antioxidant capacity in maize kernels and also to the hydrophilic fraction, while the lipophilic fraction was not related to antioxidant capacity and visual selection for kernel color increased anthocyanin content. They also concluded that the traditional method for making maize bread out of whole maize flour has not significant effects on pigment content or antioxidant capacity. Another breeding objective is grain health, particularly, genetic variability for mycotoxin contamination has been reported in maize, and there are selection programs under way (167, 168). Maize breeding has primarily focused on increasing stability and grain yield potential, under abiotic and biotic stresses (169). In the last decade, however, much effort has been made in evaluating and using the diversity of maize also on the improvement of animal feed and human nutrition (169). Currently, maize breeding efforts for improved chemical composition is being extended beyond the traditional targets of starch, oil, and protein to include components such as vitamins, and antioxidant secondary metabolites with considerable consequences for human health (170). By using marker-assisted selection, a few nutritional trait-associated genes or quantitative trait loci (QTLs) for maize protein quality, oil content and provitamin A levels have been introgressed into elite maize lines for their quality improvement (170).

The difficulty of tracking particular quality compounds using traditional breeding methodologies and the importance these compounds have on consumers' food acceptance, demonstrated the need of using other approaches such as marker-assisted selection (MAS) to breed for such traits. Taking advantage of a collection of maize inbred lines in which a considerable amount of the unexplored Portuguese maize germplasm is present, several genome wide association companion studies took place, to develop more efficient breeding tools to support the improvement of the difficult-to-handle complex organoleptic, nutritional and processing quality traits (171–173). These studies identified 64 genomic regions associated with 15 different volatiles (173), 57 associated with 11 different traits affecting maize kernel composition (protein, fiber, fat, and starch content) and flour *breadability* (starch pasting properties and flour's mean particle size) (171) and 67 associated with color and antioxidant-related traits (carotenoids, tocopherols, and phenolic compounds) (172). The strongest single nucleotide polymorphism (SNP)-trait associations and the SNP alleles with larger effect sizes were set as priority for future validation studies. The validation needed before routine use on breeding may be attained by sequencing the associated regions in an independent germplasm such as the maize populations, which are in fact the materials typically used for the production of maize bread.

Improving maize grain for increasing provitamin A and vitamin E contents, e.g., β-carotene and α-tocopherol, as the most important bioavailable compounds for humans, is a significant issue nowadays. Besides increase of single nutrient content, simultaneous breeding for multinutritional traits have numerous beneficial effects. In the study Andelković et al. (174) micronutrient content in three colored grain populations, five inbred lines and their crosses were evaluated. Population with dark orange grain had high content of both, β-carotene (26.9 μg/g d.m.) and α-tocopherol (27.2 μg/g d.m.), and could be used as a good source for multinutrient biofortification. Development of maize genotypes enhanced with micronutrients could be achieved by development of inbred lines or creation of synthetic populations, depending on available genetic resources, potential utilization, and end-users needs.

New breeding techniques (NBTs), such as genetic transformation, can be used for breeding specific aspects of maize bread; Grosset et al. (175) reported that the introduction of a gene coding for a barley CMd protein into immature maize kernels by micro-projectile bombardment was successful as the gene was expressed in the outermost cell layers of maize endosperm in both a tissue-specific and a developmentally determined manner.

## Conclusions

Based on the large published literature and wide variety of research lines (Table 1), in order to fulfill social demands, current maize research and breeding should focus on supporting sustainable production of healthy food with high quality and safety. As maize is a major crop with a large potential of breeding and adaptation, the main objectives of research and breeding include releasing varieties with adaptation to local conditions and high added value given by quality parameters and health interests, such as large antioxidant capacity, resistance to micotoxin contamination, or quality gluten-free products. Food safety is also a concern due to the increase of contamination and the reduction of plant biodiversity in agricultural fields and the surrounding natural areas. Actually, maize has outstanding ability of bioaccumulating heavy metals (i.e., Cd and Zn), and has been proposed as natural decontaminant from heavy metals (176, 177), but this ability could hinder food safety.

**Table 1:** Research lines about traditional maize food in Europe and references for each line.

**Type**	**References**	
Bread	Making process	(17–19, 24)
	Food technologies	(20–23, 26–30, 58, 59, 94–97, 107, 123, 124, 146)
	Genetic diversity	(16–18, 20, 57–61, 98, 107, 125, 126, 134)
	Nutrition and health	(16, 20, 55, 56, 61, 73, 74, 79, 80, 96–98, 123–126, 134, 169, 170)
	Quality	(20, 59, 60, 146, 148, 149, 153, 154, 158)
	Breeding	(9, 10, 16, 18, 161–164, 167–175)
Italian polenta	Making process	(31, 32)
	Food technologies	(33, 64, 66)
	Genetic diversity	(33, 62–66, 89, 90, 140)
	Nutrition and health	(65, 66, 88–90, 93, 116–118, 139)
Western specialties Talo and Gofio	Making process	(34, 40, 44)
	Food technologies	(38, 45–47)
	Genetic diversity	(35–37)
	Nutrition and health	(41)
Eastern specialties and cookies	Food technologies	(67, 83)
	Genetic diversity	(67–71)
	Nutrition and health	(67–71, 83, 85)

Therefore, breeding efforts to develop maize varieties for making bread still require establishing clear criteria for measuring quality of maize kernel, developing sustainable agricultural and post-harvest management practices and advanced breeding materials to minimize health risks caused by maize grain mycotoxin contamination, and exhaustive characterization of nutritional profile of maize kernel to determine the potentiality of maize bread as functional food.

## Author Contributions

PR conceptualization and redaction of Spanish sections. MA, ID, PMM, and MCVP redaction of Portuguese sections. VA and SŽ redaction of Serbian sections. CB and RR redaction of Italian sections. JG redaction of Spanish sections. RM structuration and organization. All authors have read and agreed to the published version of the manuscript.

## Funding

This research was funded by Spanish Ministerio de Innovación y Universidades (MCIU), the Agencia Estatal de Investigación (AEI) and the European Fund for Regional Development (FEDER), UE (project code PID2019-108127RB-I00), the Xunta de Galicia-Spain (project code IN607A 2021/03), and FCT, Portugal (Research Unit UID/04551/2020).

## Conflict of Interest

The authors declare that the research was conducted in the absence of any commercial or financial relationships that could be construed as a potential conflict of interest.

## Publisher's Note

All claims expressed in this article are solely those of the authors and do not necessarily represent those of their affiliated organizations, or those of the publisher, the editors and the reviewers. Any product that may be evaluated in this article, or claim that may be made by its manufacturer, is not guaranteed or endorsed by the publisher.
